# *Fusobacterium nucleatum* promotes epithelial‐mesenchymal transiton through regulation of the lncRNA MIR4435‐2HG/miR‐296‐5p/Akt2/SNAI1 signaling pathway

**DOI:** 10.1111/febs.15233

**Published:** 2020-02-12

**Authors:** Shuwei Zhang, Chen Li, Junchao Liu, Fengxue Geng, Xiaoting Shi, Qian Li, Ze Lu, Yaping Pan

**Affiliations:** ^1^ Department of Periodontics School and Hospital of Stomatology China Medical University Shenyang China; ^2^ Liaoning Provincial Key Laboratory of Oral Diseases School and Hospital of Stomatology China Medical University Shenyang China

**Keywords:** E‐cadherin, epithelial–mesenchymal transition, *Fusobacterium nucleatum*, MIR4435‐2HG, SNAIL1

## Abstract

*Fusobacterium nucleatum*, an anaerobic oral opportunistic pathogen associated with periodontitis, has been considered to be associated with the development of oral squamous cell carcinoma (OSCC). However, the initial host molecular alterations induced by *F. nucleatum* infection which may promote predisposition to malignant transformation through epithelial–mesenchymal transition (EMT) have not yet been clarified. In the present study, we monitored the ability of *F. nucleatum* to induce EMT‐associated features, and our results showed that *F. nucleatum* infection promoted cell migration in either noncancerous human immortalized oral epithelial cells (HIOECs) or the two OSCC cell lines SCC‐9 and HSC‐4, but did not accelerate cell proliferation or cell cycle progression. Mesenchymal markers, including N‐cadherin, Vimentin, and SNAI1, were upregulated, while E‐cadherin was decreased and was observed to translocate to the cytoplasm. Furthermore, FadA adhesin and heat‐inactivated *F. nucleatum* were found to cause a similar effect as the viable bacterial cells. The upregulated lncRNA MIR4435‐2HG identified by the high‐throughput sequencing was demonstrated to negatively regulate the expression of miR‐296‐5p, which was downregulated in *F. nucleatum*‐infected HIOECs and SCC‐9 cells. The binding of MIR4435‐2HG and miR‐296‐5p was validated via a dual‐luciferase reporter assay. Additionally, knockdown of MIR4435‐2HG with siRNA leads to a decrease in SNAI1 expression, while miR‐296‐5p could further negatively and indirectly regulate SNAI1 expression via Akt2. Therefore, our study demonstrated that *F. nucleatum* infection could trigger EMT via lncRNA MIR4435‐2HG/miR‐296‐5p/Akt2/SNAI1 signaling pathway, and EMT process may be a probable link between *F. nucleatum* infection and initiation of oral epithelial carcinomas.

## Graphical abstract

*Fusobacterium nucleatum *is an anaerobic oral opportunistic pathogen; its precise role in inducing epithelial–mesenchymal transition (EMT) has not been clarified. Here, we reported that* F. nucleatum* upregulated the expression of MIR4435‐2HG, which could specifically bind with miR‐296‐5p, weakening its ability to silence Akt2. In turn, this could then activate SNAI1 expression and eventually contribute to EMT in the infected oral epithelial cells.

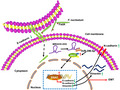

**Abbreviations**
Akt2: Akt serine/threonine kinase 2
EMT: epithelial–mesenchymal transition
*Fn*: *Fusobacterium nucleatum*
GF: gingival fibroblast
HIOECs: human immortalized oral epithelial cells
HNSCC: head and neck squamous cell carcinoma
KEGG: Kyoto Encyclopedia of Genes and Genomes
MOI: multiplicity of infection
OSCC: oral squamous cell carcinoma
*Sg*: *Streptococcus gordonii*
SLUG: snail family transcription repressor 2
SNAI1: snail family transcription repressor 1
TWIST1: twist basic helix‐loop‐helix transcription factor 1
ZEB1: zinc finger E‐Box binding homeobox 1

## Introduction

Periodontitis is the sixth most prevalent disease worldwide and has been linked to oral squamous cell carcinoma (OSCC) 1, 2. *F. nucleatum* is an anaerobic periodontal pathogen acting as the ‘bridge bacterium’ that links early and late colonizers, for instance, *Streptococcus gordonii* and *Porphyromonas gingivalis* in plaque biofilm 3. A link between *F. nucleatum* and cancer was first established upon detection of the abundance of *F. nucleatum* in colorectal cancer patients using metagenomics methods 4. To date, extensive researches have explored the contribution of *F. nucleatum* to the development of colorectal carcinomas 4, 5, 6. A significant abundance of *F. nucleatum* has also been detected in patients with OSCC 7, 8, 9. In line with the previous studies, our recent study revealed that *F. nucleatum* was present at a higher level in OSCC tissues than in normal tissues by analyzing 61 oral cancer tissues and their adjacent paracancerous tissues as well as 30 normal tissues using 16S rRNA amplicon sequencing and qPCR 10. However, the regulatory role of *F. nucleatum* in malignant transformation or oncogenic progression of oral epithelial cells remains largely unknown.

Epithelial–mesenchymal transition (EMT) was defined as a rapid and often reversible alteration from epithelial to mesenchymal cell phenotype with weakened cell–cell junctions and remodeling of the cytoskeleton 11. The definition of EMT has now been broadened based on many observations, and a partial EMT has been associated with cancer development, wound healing, fibrosis, and cancer progression 12, 13. The coexpression of epithelial and mesenchymal markers is often used to define the hybrid state 12. A cluster of pleiotropic transcription factors has been demonstrated to orchestrate EMT programs, including SNAI1, SLUG, ZEB, and TWIST1, which can upregulate the mesenchymal markers Vimentin and N‐cadherin and ultimately repress the expression of E‐cadherin, which is a hallmark of the epithelial state 14, 15.

Noncoding RNAs with limited protein‐coding capacity have emerged as crucial regulators of EMT. Based on high‐throughput sequencing and biological techniques, an increasing number of new microRNAs, lncRNAs, and circRNAs are being uncovered, and their pivotal roles in regulating EMT have been extensively investigated 16. HOX transcript antisense RNA (HOTAIR) is frequently overexpressed in a wide variety of malignancies and has been demonstrated to enhance EMT by sponging miR‐23b‐3p from ZEB1 in hepatocellular carcinoma 17. Zhang *et al*. 18 reported that circRNA SMAD2 could inhibit EMT by targeting miR‐629 in hepatocellular carcinoma. MIR4435‐2HG, also known as LINC00978, is a novel lncRNA located in 2q13 and has been reported to promote cell growth and tumorigenesis in lung and gastric cancers 19.

In this research, we uncovered the precise role of *F. nucleatum* in the induction of EMT in oral epithelial cells, as evidenced by promoted cell migration, upregulated expression of N‐cadherin, Vimentin, and SNAI1 and functional loss of E‐cadherin. Our results demonstrated that *F. nucleatum* infection upregulated the expression of MIR4435‐2HG, which could specifically bind with miR‐296‐5p to downregulate its expression level, weakening the ability of miR‐296‐5p to silence its target gene Akt2, which could then activate the expression of SNAI1, and eventually contribute to EMT in the infected oral epithelial cells. Taken together, this study suggests a novel mechanism by which *F. nucleatum* can contribute to EMT and potentially drive the progression of oral cancer.

## Results

### High abundance of *F. nucleatum* in clinical samples

**Figure 1:**
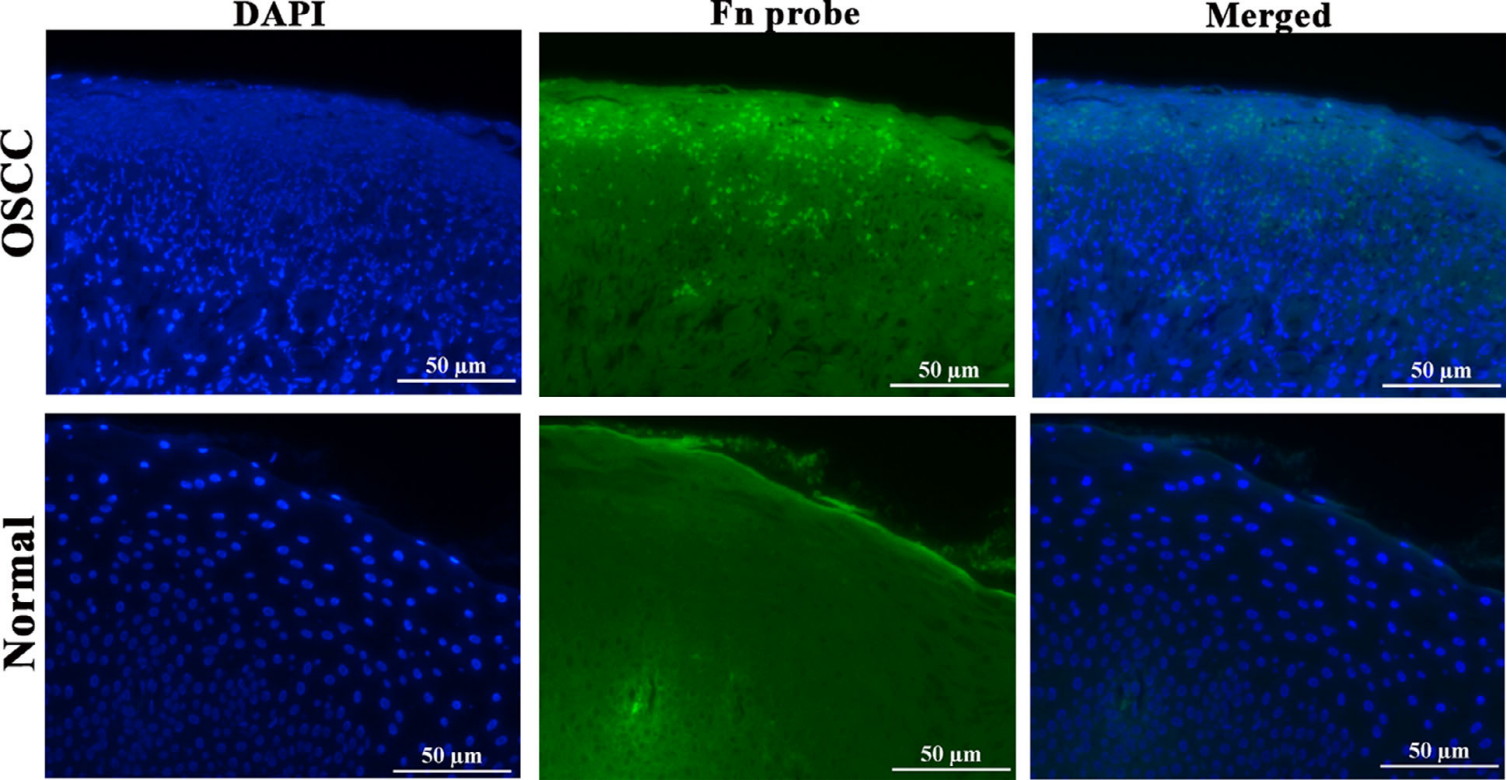
*Fusobacterium nucleatum* was present in OSCC. High enrichment of *F. nucleatum* in OSCC tissues was detected by FISH using Alexa Fluor 488‐labeled *F. nucleatum*‐specific probe (green). Cell nuclei were stained with DAPI (blue) (*n* = 10 samples, each group). Scale bar represents 50 μm. Magnification, 200×.

Oral squamous cell carcinoma samples (*n* = 10) and normal oral tissues (*n* = 10) were histopathologically confirmed by hematoxylin and eosin staining (data not shown). Fluorescence in situ hybridization (FISH) using Alexa Fluor 488‐labeled *F. nucleatum*‐specific probe was conducted to validate the existence of *F. nucleatum* in oral tumor species and the normal tissues. As shown in Fig. 1, *F. nucleatum* was highly abundant in OSCC species and was observed mainly within the epithelium, including the superficial and deep layers. On the contrary, fewer *F. nucleatum* was observed in the normal tissues.

### *F. nucleatum* infection did not change oral epithelial cells proliferation or cell cycle progression but promoted cell apoptosis and migration

**Figure 2:**
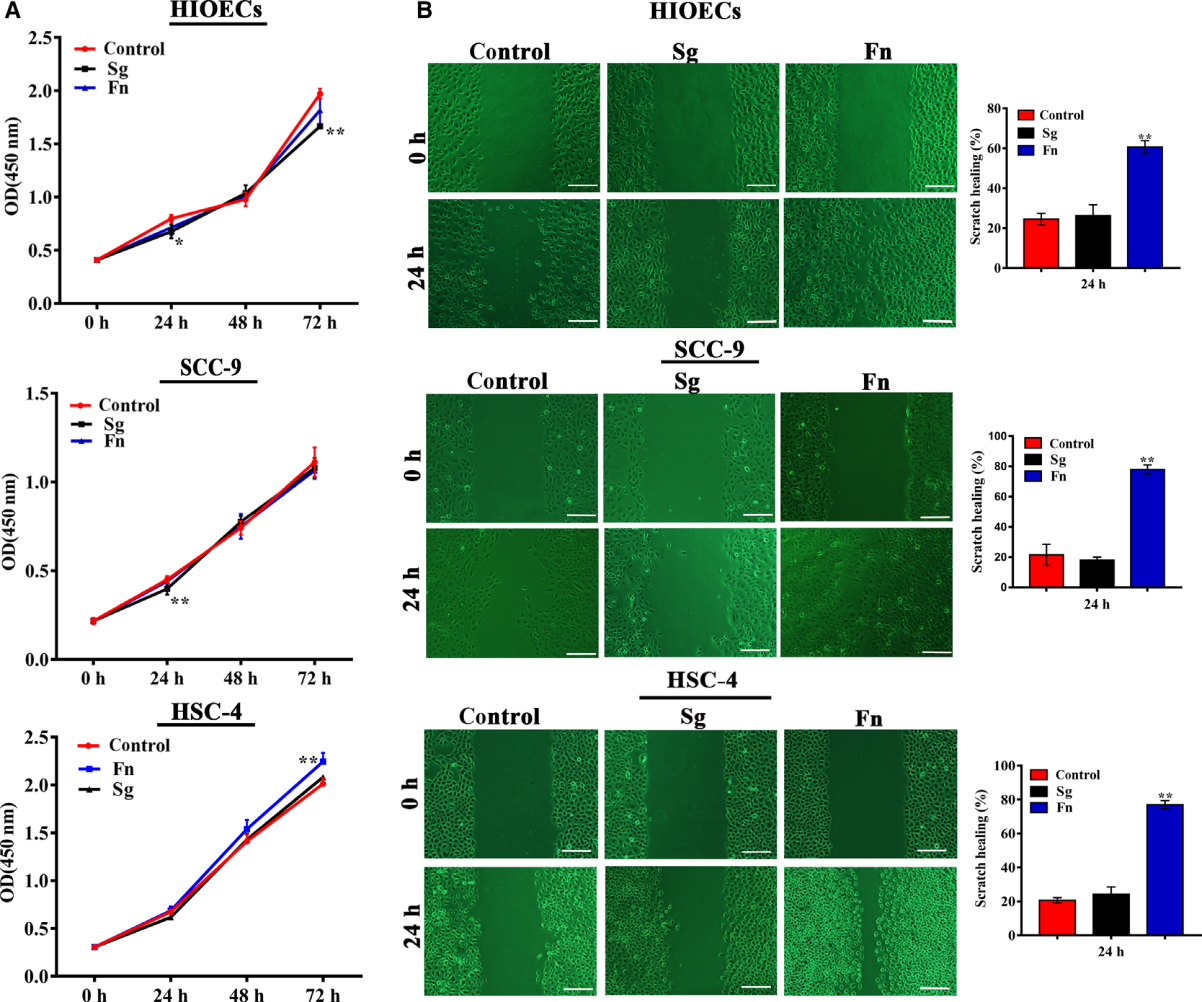
Effect of *Fusobacterium nucleatum* or *Streptococcus gordonii* infection on cell proliferation and cell migration. HIOECs, SCC‐9, and HSC‐4 cells were infected with *F. nucleatum* or *S. gordonii* at a MOI of 100 : 1, (A) cell proliferation was assayed by CCK‐8, and (B) cell migration was evaluated by scratch assay. Scale bar represents 200 μm. The data are presented as the mean ± SD obtained from three independent experiments (*n* = 3). *P < 0.05, **P < 0.01 vs. the control cells (Student's *t‐*test).

The results of CCK‐8 assay showed that neither *F. nucleatum* nor *S. gordonii* infection affected cell proliferation significantly in comparison with the uninfected cells (Fig. 2A). Similarly, *F. nucleatum* infection did not accelerate the cell cycle in either human immortalized oral epithelial cells (HIOECs) or SCC‐9 cells (Fig. S1A). The apoptosis rates of *F. nucleatum* **‐**infected HIOECs (53.3 ± 18.61%) and SCC‐9 cells (36.67 ± 11.29%) were much higher than those in noninfected HIOECs (11.03 ± 3.31%) and SCC‐9 cells (5.9 ± 3.19%) (Fig. S1B). As shown in Fig. 2B, *F. nucleatum* significantly accelerated the cell migration compared with *S. gordonii*‐infected and the control cells. Further, the activity of MMP‐9 and MMP‐2 following *F. nucleatum* infection was corroborated by zymography (Fig. S2). As shown, the activity of MMP‐9 secreted by *F. nucleatum*‐infected cells was significantly increased compared with the noninfected controls. However, no obvious increase of MMP‐2 was detected in *F. nucleatum*‐infected cells.

### *F. nucleatum* infection promoted the expression of mesenchymal markers and caused E‐cadherin translocation

The impact of *F. nucleatum* on SNAI1 expression in HIOECs was investigated using qRT‐PCR. As shown in Fig. S3, *F. nucleatum* increased SNAI1 mRNA levels in a dose‐dependent manner, with maximal induction occurring with a multiplicity of infection (MOI) of 100. An increase in the amount of SNAIL1 protein was also observed at 24 h following *F. nucleatum* infection, whereas *S. gordonii* did not cause elevated SNAIL1 protein expression (Fig. S4), which indicates that the mesenchymal changes in the infected cells are *F. nucleatum*‐specific, while *S. gordonii* did not exert the same effect.

**Figure 3:**
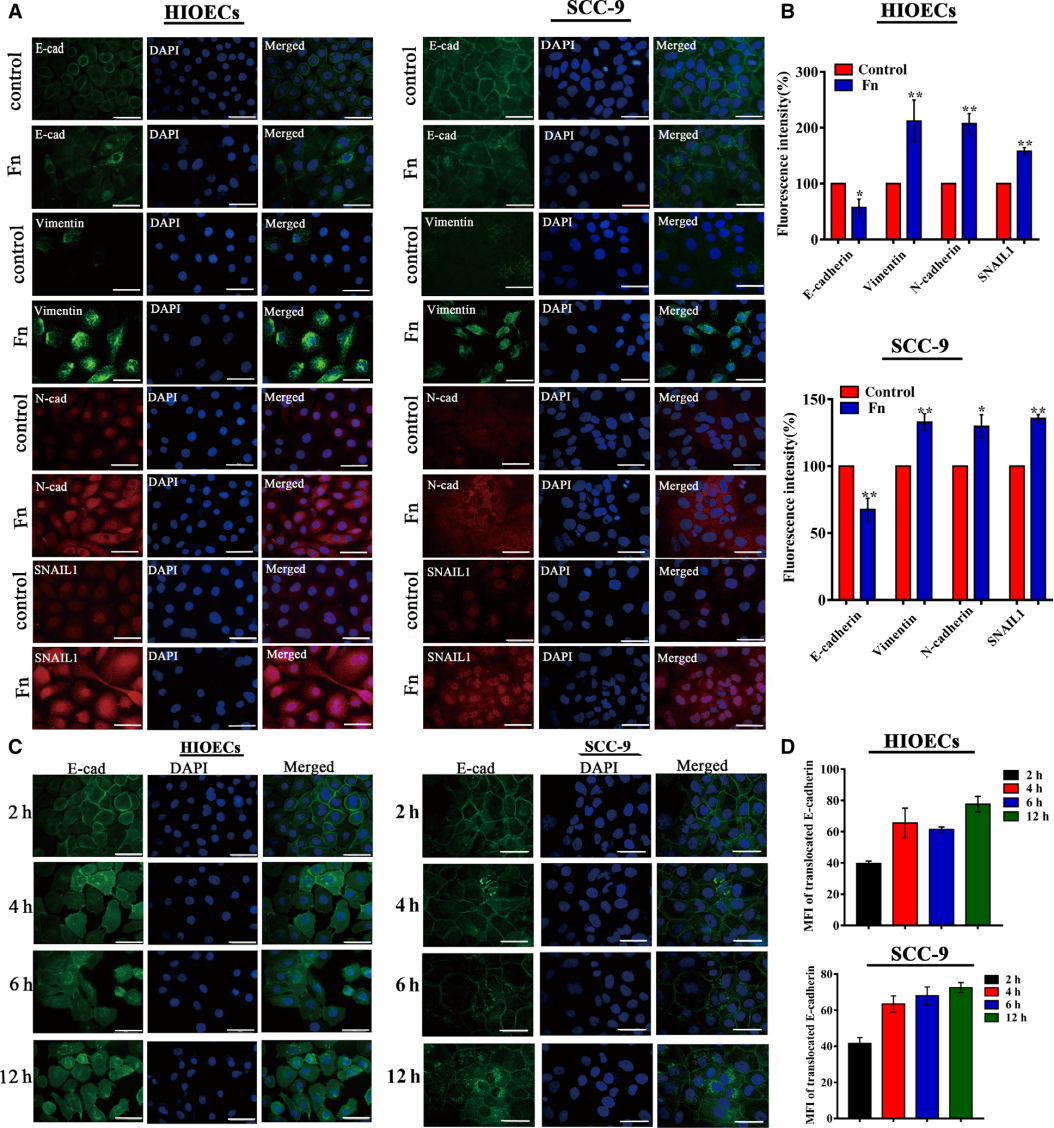
Immunofluorescence of the EMT markers. (A, B) Representative images of the expression of E‐cadherin, Vimentin, N‐cadherin, and SNAIL1 visualized using immunofluorescence staining. HIOECs and SCC‐9 cells were infected with *Fusobacterium nucleatum* at a MOI of 100 : 1 for 24 h, and the fluorescent levels were analyzed using imagej software (NIH, Bethesda, MD, USA). *F. nucleatum* infection increased the expression of Vimentin, N‐cadherin, and SNAIL1 but induced the loss of E‐cadherin at the cell membrane compared with the control cells. Data are presented as mean ± SD from three independent experiments (*n* = 3). *P < 0.05, **P < 0.01 vs. the control cells (Student's *t‐*test). (C, D) Representative immunofluorescent images of E‐cadherin location in *F. nucleatum*‐infected HIOECs and SCC‐9 cells at 2–12 h, and the mean fluorescence intensity of translocated E‐cadherin were analyzed. MOI = 100 : 1. Data are presented as mean ± SD from three independent experiments (*n* = 3, each group). Scale bar represents 50 µm.

**Figure 4:**
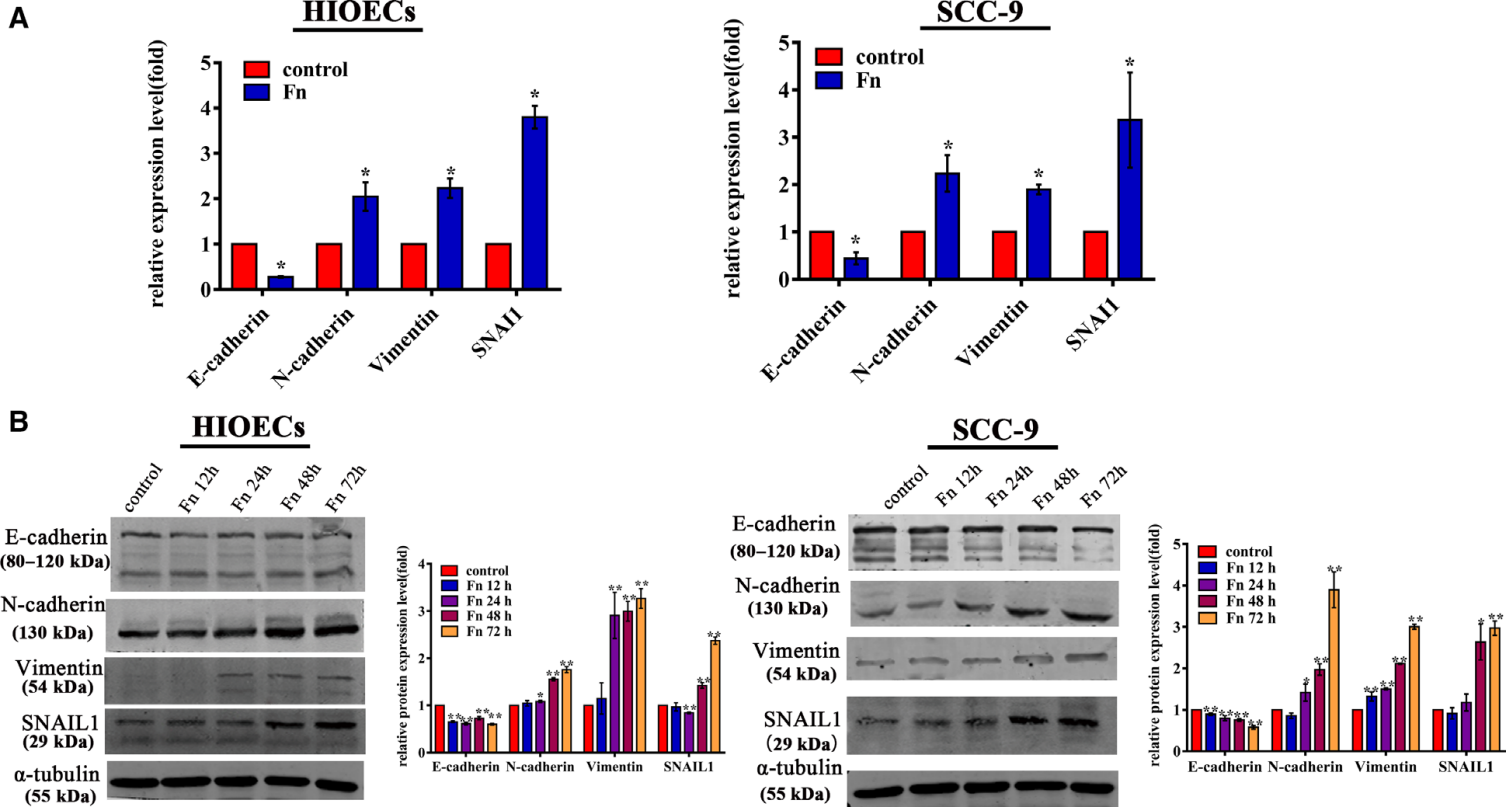
The mRNA and protein expressions of EMT markers. HIOECs and SCC‐9 cells were infected with *Fusobacterium nucleatum* at a MOI of 100 : 1, and (A) the mRNA and (B) protein expressions were assayed by qRT‐PCR and western blotting. The data are presented as the mean ± SD obtained from three independent experiments (*n* = 3). *P < 0.05, **P < 0.01 vs. the control cells (Student's *t‐*test).

The expression of E‐cadherin, Vimentin, N‐cadherin, and SNAIL1 in *F. nucleatum* **‐**infected HIOECs and SCC‐9 cells at 24 h was visualized by immunofluorescence staining. As shown in Fig. 3, E‐cadherin was decreased, while Vimentin, N‐cadherin, and SNAIL1 proteins were increased in *F. nucleatum*‐infected HIOECs and SCC‐9 cells, which were consistent with the qRT‐PCR and western blotting results (Fig. 4). Surprisingly, the micrographs of fluorescently stained HIOECs and SCC‐9 cells revealed E‐cadherin translocation from the membrane to the cytoplasm at 24 h postinfection. As illustrated in Fig. 3A,B, E‐cadherin was mainly distributed on the cell membrane in noninfected HIOECs and SCC‐9 cells as a component of the adherens junction complexes, while it appeared disorganized and punctiform in the cytoplasm of the *F. nucleatum‐*infected cells. Additionally, as the infection time extended from 2 to 12 h, increasing E‐cadherin shifted from the membrane to the cytoplasm in *F. nucleatum‐*infected HIOECs and SCC‐9 (Fig. 3C,D). Additionally, purified *F. nucleatum* adhesin FadA was capable of regulating the expression of the above EMT markers, and heat‐inactivated *F. nucleatum* showed similar effects on EMT markers in both HIOECs and SCC‐9 cells (Fig. S5).

### Differentially expressed transcriptomic genes and validation of certain selected genes by qRT‐PCR

**Figure 5:**
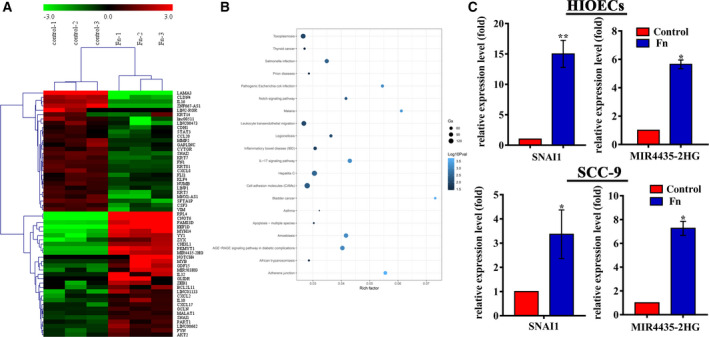
Sequencing data analysis and gene expression validation by qRT‐PCR. (A) Hierarchical clustering analysis of differentially expressed genes associated with EMT and inflammation in *Fusobacterium nucleatum*‐infected HIOECs. MOI = 100 : 1. The selected genes are shown in a tree view with RNA expression from low (green) to high (red). (B) KEGG pathway analysis revealed that the selected genes were enriched in cell adhesion signaling. (C) The expression levels of MIR4435‐2HG and SNAI1 were validated by qRT‐PCR. MIR4435‐2HG and SNAI1 were upregulated significantly compared with those in the control cells. MOI = 100 : 1. Data are shown as the mean ± SD from three independent experiments (*n* = 3). *P < 0.05, **P < 0.01 vs. the control cells (Student's *t‐*test).

According to the high‐throughput sequencing data, 887 mRNAs were upregulated, while 1866 mRNAs were downregulated in *F. nucleatum*‐infected cells. Additionally, 74 upregulated and 90 downregulated lncRNAs were identified. Aberrantly expressed genes, including a majority of mRNAs and certain lncRNAs associated with EMT, were clustered and are shown in Fig. 5A. Kyoto Encyclopedia of Genes and Genomes (KEGG) pathway analysis indicated that *F. nucleatum* infection might activate signaling pathways involved in cell adhesion (Fig. 5B). The selected differentially expressed genes were validated by qRT‐PCR. In both *F. nucleatum*‐infected HIOECs and SCC‐9 cells, SNAI1 and MIR4435‐2HG were significantly increased (Fig. 5C), while other transcription factors such as ZEB2, TWIST1, and SLUG were not significantly affected in response to *F. nucleatum* infection (Fig. S6). Other differentially expressed genes are displayed in Fig. S7, and their expression levels overall correspond with the sequencing data.

### MIR4435‐2HG was upregulated in *F. nucleatum*‐infected HIOECs and SCC‐9 cells and regulated the expression of SNAIL1

**Figure 6:**
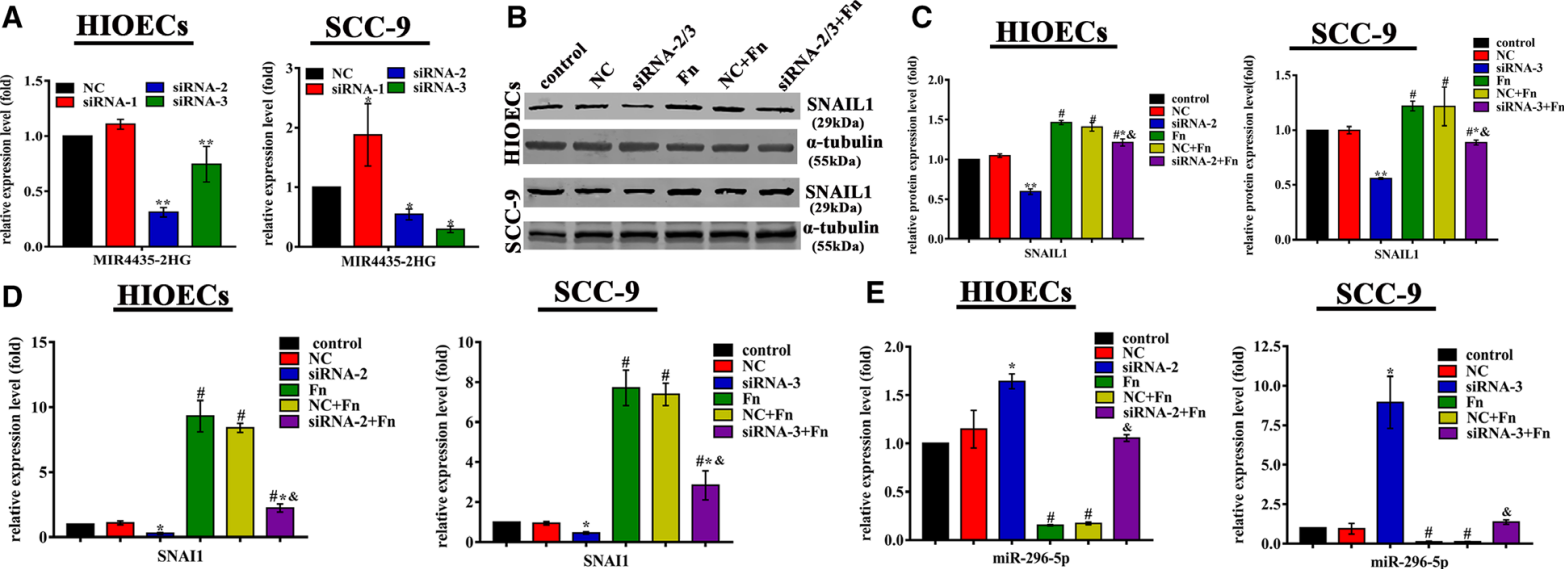
MIR4435‐2HG affected the expression of SNAI1 and miR‐296‐5p. (A) HIOECs and SCC‐9 cells were transfected with siRNAs against MIR4435‐2HG or si‐NC, and the relative expression of MIR‐4435‐2HG was determined by qRT‐PCR. Data are shown as the mean ± SD from three independent experiments (*n* = 3). *P < 0.05, **P < 0.01 vs. NC (Student's *t‐*test). (B–D) MIR4435‐2HG knockdown suppressed the expression of SNAI1 in both HIOECs and SCC‐9 cells, and *Fusobacterium nucleatum* rescued SNAI1 expression after MIR4435‐2HG knockdown measured by western blotting and qRT‐PCR. MOI = 100 : 1. Data are shown as the mean ± SD from three independent experiments (*n* = 3). *P < 0.05 and **P < 0.01 vs. the NC group. ^#^ *P* < 0.05 vs. the control group and ^&^ *P* < 0.05 vs. the NC + *Fn* group (one‐way ANOVA). (E) Relative expression of miR‐296‐5p determined by qRT‐PCR. miR‐296‐5p was decreased in *F. nucleatum*‐infected HIOECs and SCC‐9 cells, while MIR4435‐2HG knockdown specifically upregulated the expression of miR‐296‐5p. MOI = 100 : 1. Data are presented as the mean ± SD from three independent experiments (*n* = 3). *P < 0.05 and **P < 0.01 vs. the NC group. ^#^ *P* < 0.05 vs. the control group and ^&^ *P* < 0.05 vs. the NC + *Fn* group (one‐way ANOVA).

Among the upregulated genes, a novel lncRNA MIR4435‐2HG, which has been seldom investigated in oral epithelial cells, aroused our interest. The expression levels of MIR4435‐2HG and SNAI1 were found to be positively correlated in various carcinomas, including head and neck squamous cell carcinoma (HNSCC) in the online database TCGA‐Pan‐Cancer (ChIPBase v2.0) (Fig. S8). siRNA transfection was conducted to investigate the regulation of MIR4435‐2HG on SNAI1 expression. The effective knockdown of MIR4435‐2HG was determined by qRT‐PCR, and siRNA‐2 for HIOECs and siRNA‐3 for SCC‐9 cells were used in the subsequent study because of their highest silencing efficiency (Fig. 6A). The expression of SNAI1 was inhibited in MIR4435‐2HG‐silenced HIOECs and SCC‐9 cells compared with that in cells transfected with si‐NC (Fig. 6B–D). HIOECs and SCC‐9 cells with attenuated MIR4435‐2HG expression were then infected with *F. nucleatum* at a MOI of 100 : 1 over 24 h, and the results showed that knockdown of MIR4435‐2HG prevented the *F. nucleatum*‐induced increase in SNAI1 expression (Fig. 6B–D).

### MIR4435‐2HG competitively bound miR‐296‐5p and regulated miR‐296‐5p negatively and directly

The assumption that MIR4435‐2HG acts as a miRNA sponge to positively regulate SNAI1 expression was first taken into consideration. Putative microRNAs that could bind with the MIR4435‐2HG sequence were predicted using DIANA Tools (LncBase v.2). MicroRNAs that might target SNAI1 were predicted by miRDB, and the overlapping miRNAs were obtained. Among the predicted microRNAs, miR‐296‐5p was decreased in *F. nucleatum*‐infected HIOEC (Fig. S9), and miR‐296‐5p was previously reported to be associated with EMT 20. Therefore, miR‐296‐5p was chosen as a candidate gene for the subsequent study. As expected, the qRT‐PCR results showed that miR‐296‐5p was downregulated in *F. nucleatum*‐infected HIOECs and SCC‐9 cells but significantly elevated in MIR4435‐2HG‐silenced HIOECs and SCC‐9 cells. Moreover, MIR4435‐2HG deficiency impeded the *F. nucleatum*‐induced decrease in miR‐296‐5p expression (Fig. 6E).

**Figure 7:**
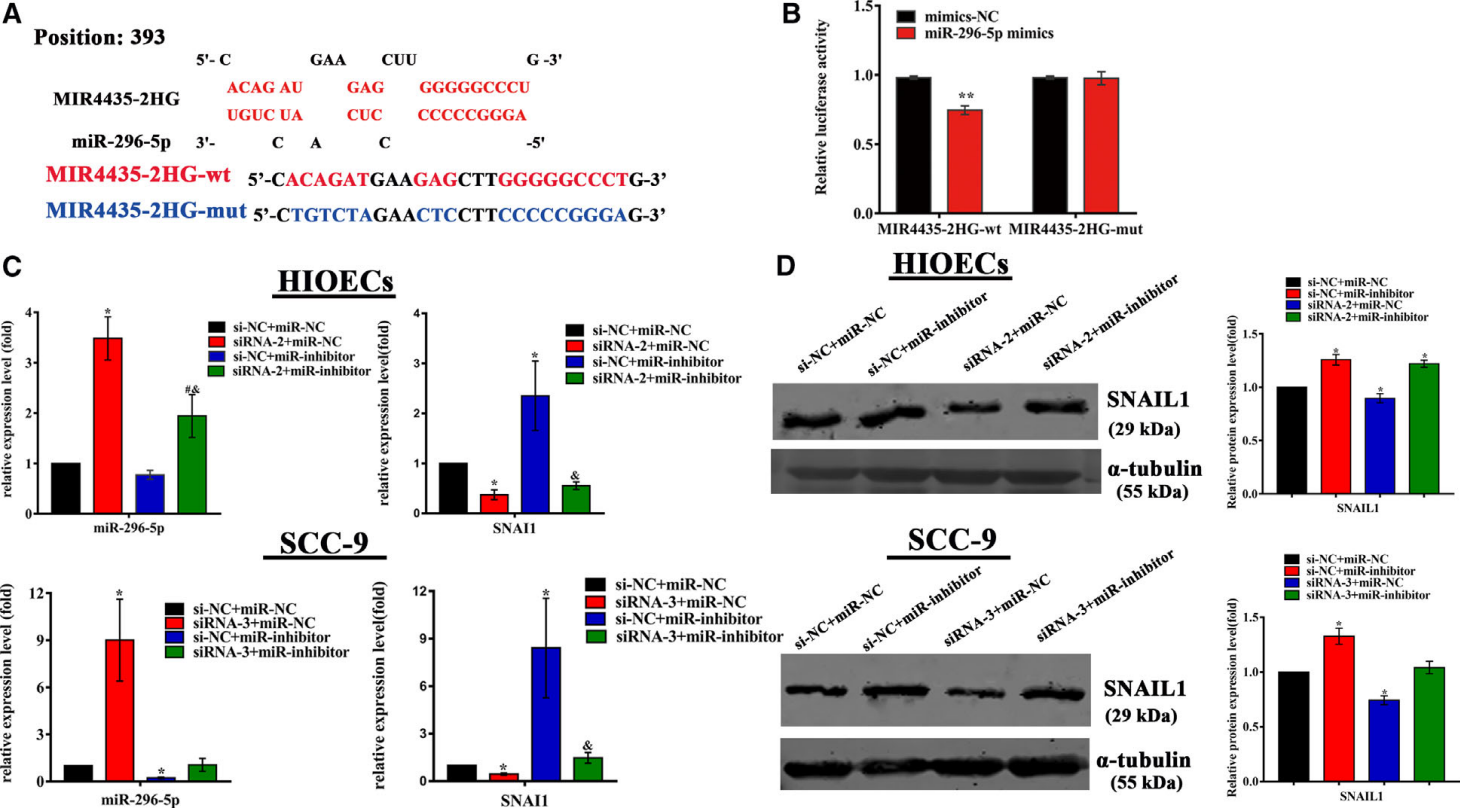
MIR4435‐2HG affected the expression of SNAI1 via regulation of miR‐296‐5p. (A) The predicted miR‐296‐5p binding site in the MIR4435‐2HG sequence. Red indicates the complementary nucleotides. The sequences of wild‐type MIR4435‐2HG (red) and the mutant MIR4435‐2HG (blue) are also listed. (B) A dual‐luciferase reporter gene assay was performed to identify the interaction between MIR4435‐2HG and miR‐296‐5p in HEK 293T cells cotransfected with MIR4435‐2HG‐wt or MIR4435‐2HG‐mut and miR‐296‐5p mimics or mimics‐NC. Data are presented as the mean ± SD obtained from three independent experiments (*n* = 3). **P < 0.01 vs. MIR4435‐2HG‐wt + mimics‐NC group (Student's *t‐*test). (C, D) Relative expression of miR‐296‐5p and SNAIL1 in HIOECs and SCC‐9 cells following cotransfection of siRNAs or si‐NC targeting MIR4435‐2HG and miR‐296‐5p inhibitor or miR‐NC. Data are presented as the mean ± SD obtained from three independent experiments (*n* = 3). *P < 0.05 vs. the si‐NC + miR‐NC group, ^&^ *P* < 0.05 vs. the si‐NC + miR‐inhibitor group (one‐way ANOVA).

A dual‐luciferase reporter assay was performed to verify MIR4435‐2HG binding to miR‐296‐5p according to the predicted binding sites (Fig. 7A). When MIR4435‐2HG vectors were cotransfected with miR‐296‐5p mimics, the luciferase activity of the MIR4435‐2HG‐wt vector was notably suppressed, while no significant change was observed in the luciferase activity of the MIR4435‐2HG‐mut vector compared with that of the control group (Fig. 7B).

### MiR‐296‐5p downregulated the expression of SNAI1 by targeting Akt2

Based on the information obtained from miRDB, SNAI1 might be a target of miR‐296‐5p. The abovementioned results showed that miR‐296‐5p was downregulated while SNAI1 was upregulated in *F. nucleatum*‐infected HIOECs and SCC‐9 cells (Fig. 6B–E). To decipher the regulation of miR‐296‐5p on SNAI1 expression, HIOECs and SCC‐9 cells were transfected with miR‐296‐5p inhibitor, and SNAI1 was shown to be significantly upregulated compared with that in cells transfected with miR‐NC (Fig. 7C,D). In HIOECs and SCC‐9 cells cotransfected with siRNAs targeting MIR4435‐2HG and miR‐296‐5p inhibitor, the expression of SNAI1 was reduced compared with cells cotransfected with si‐NC and miR‐296‐5p inhibitor (Fig. 7C,D). However, a dual‐luciferase reporter assay showed that miR‐296‐5p did not bind with the 3′UTR of SNAI1 directly (Fig. S10).

**Figure 8:**
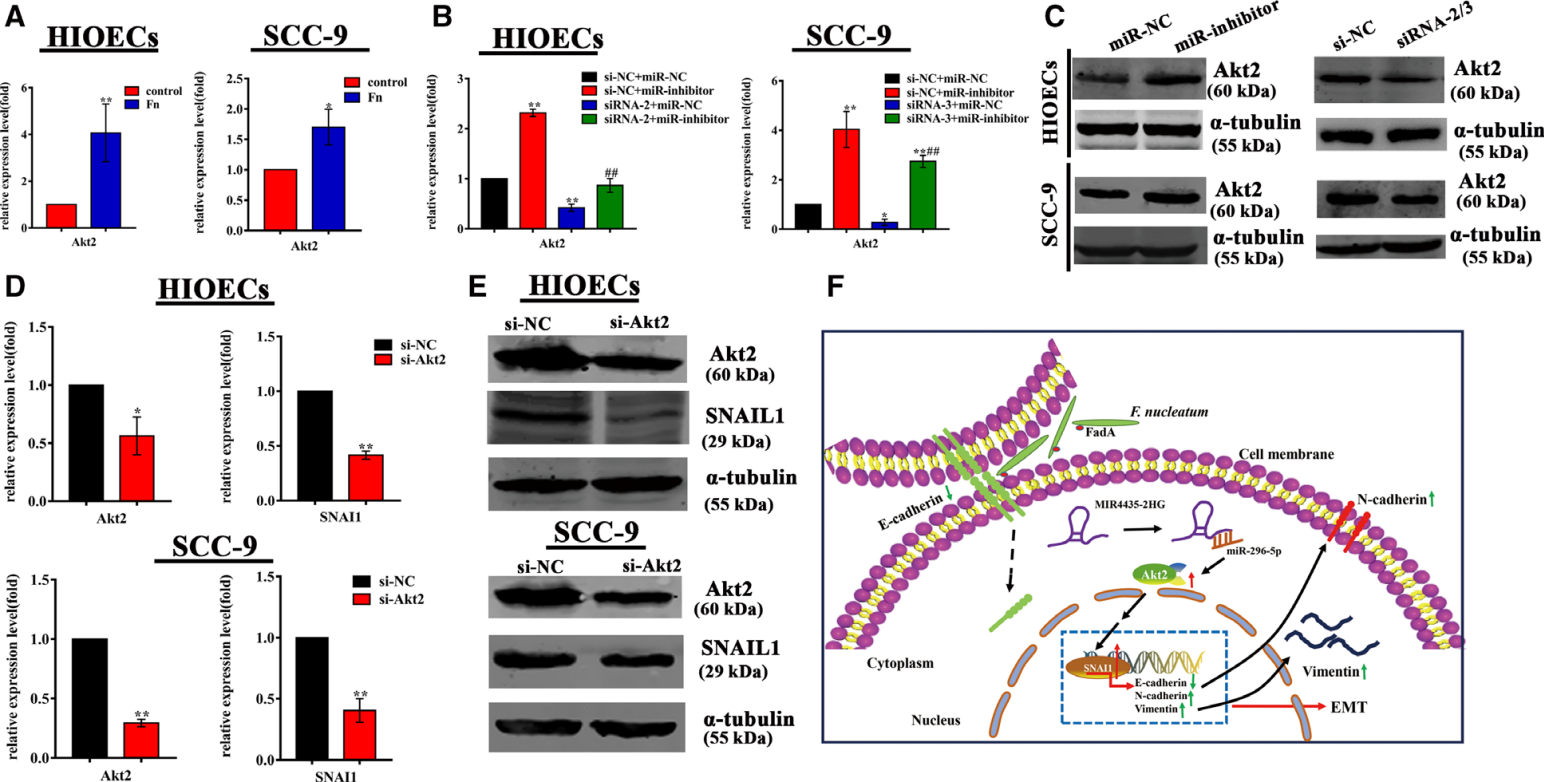
Akt2 knockdown inhibited SNAI1 expression. (A) Akt2 was upregulated in *Fusobacterium nucleatum*‐infected HIOECs and SCC‐9 cells as assayed by qRT‐PCR. MOI = 100 : 1. The data are presented as the mean ± SD obtained from three independent experiments (*n* = 3). *P < 0.05, **P < 0.01 vs. the control cells (Student's *t‐*test). (B, C) Relative expression of Akt2 and SNAI1 in HIOECs and SCC‐9 cells following cotransfection of siRNAs or si‐NC targeting MIR4435‐2HG and miR‐296‐5p inhibitor or miR‐NC assayed by qRT‐PCR and western blotting. The data are presented as the mean ± SD obtained from three independent experiments (*n* = 3). *P < 0.05, **P < 0.01 vs. the si‐NC + miR‐NC group, ^##^ *P* < 0.01 vs. the si‐NC + miR‐inhibitor group (one‐way ANOVA). (D, E) Akt2 knockdown inhibited the expression of SNAI1 detected by qRT‐PCR and western blotting. The data are presented as the mean ± SD obtained from three independent experiments (*n* = 3). *P < 0.05, **P < 0.01 vs. si‐NC cells (Student's *t‐*test). (F) A schematic diagram of the proposed EMT mechanism based on the present model. *F. nucleatum* upregulates MIR4435‐2HG, which competitively binds miR‐296‐5p and weakens the inhibiting effect of miR‐296‐5p on SNAI1 indirectly via its direct targeting of Akt2. As a dominant transcription factor, upregulated SNAI1 suppresses the expression of E‐cadherin and promotes the expression of mesenchymal markers such as N‐cadherin and Vimentin.

The results of high‐throughput sequencing and qRT‐PCR showed that Akt2 was upregulated in HIOECs and SCC‐9 cells infected with *F. nucleatum* (Fig. 8A). Akt2 was downregulated in cells transfected with siRNA targeting MIR4435‐2HG and upregulated when miR‐296‐5p was inhibited. The change in Akt2 was similar to that in SNAI1 (Fig. 8B,C), and Akt2 knockdown inhibited the expression of SNAI1 (Fig. 8D,E).

## Discussion

Epithelial–mesenchymal transition is a multifaceted process involved in embryogenesis, wound healing, tissue regeneration, organ fibrosis, and tumor invasion and metastasis. Cells that undergo EMT process usually lose their epithelial characteristics and acquire mesenchymal features, including loss of cell polarity, reorganization of the cytoskeleton, weak cell–cell adhesion, and enhanced migratory capacity. This transition entails the up‐ and downregulation of different proteins responsible for a profound cellular reorganization 13. Therefore, in most experimental models, epithelial markers, such as E‐cadherin and β‐catenin, and mesenchymal markers, such as N‐cadherin, Vimentin, and fibronectin, are commonly used as indicators to confirm the occurrence of EMT 11, 12.

Recent years, microbial pathogens have been considered as EMT inducers based on their ability to modulate E‐cadherin expression via an increase in the core EMT regulatory factors. As is reported, *Helicobacter pylori* induces EMT in a cytotoxin‐associated gene A (CagA)‐dependent manner, which induces an increase in EMT markers and stabilizing SNAIL1 protein by reducing GSK‐3 activity in gastric epithelial cancer cells 21, 22, 23. Moreover, researchers have verified that FimA‐positive *P. gingivalis* was capable of initiating a mesenchymal‐like transition through ZEB1 in gingival epithelial cells 24. Of note, another study revealed that long‐term *P. gingivalis* infection strongly associates with the induction of early EMT process initiated by the phosphorylation of GSK‐3β in primary human oral epithelial cells 25. Previous work by Abdulkareem and coworkers showed that both heat‐killed *P. gingivalis* and *F. nucleatum* triggered the EMT phenotype in OSCC cells 26. *F. nucleatum*, a common inhabitant of the oral cavity, is a multifaceted bacterium that engages in diverse interactions with other oral microorganisms such as *P. gingivalis* and host cells to drive periodontal diseases 27. In the present study, elevated levels of *F. nucleatum* were observed in OSCC tissues compared with normal oral tissues, which were concordant with previous reports 28, 29. To date, few studies have focused on the role of *F. nucleatum* in EMT or the malignant transformation initiation of oral epithelial cells, and the specific mechanism has not yet been clarified. Therefore, this study is by no means intended to provide a global view on EMT. Instead, we have attempted to depict the probable signaling pathway by which *F. nucleatum* infection initiated EMT in non‐neoplastic and neoplastic oral epithelial cells.

The effects of *F. nucleatum* on cell proliferation were not quite the same. In our present study, *F. nucleatum* infection did not affect the proliferation of oral epithelial cells, which was consistent with previous study by Yu *et al*. 30 showed that *F. nucleatum* had no effect on proliferation of colorectal cancer cells HCT116 and HT29. However, *F. nucleatum* can also impair the proliferation of human gingival fibroblasts (GFs), HUVECs, or promote the proliferation of colorectal cancer cell lines LoVo and SW480 cells 31, 32, 33, 34. The discrepancy may be attributed to the cell type diversity.

Interestingly, our results showed that *F. nucleatum* increased both apoptosis and cell migration. These results are concordant with previous report that *F. nucleatum* caused the apoptosis of peripheral blood mononuclear cells and GFs 31, 35 and could also increase cell migration in human epithelial cells 36. Some explanations are possible for this phenomenon. *F. nucleatum* induced EMT in the infected oral epithelial cells, and the cells undergoing EMT acquired enhanced ability to migration supported by functional loss of E‐cadherin and elevated MMP‐9 activity. Therefore, the cells exhibited enhanced migration in spite of an increase in the apoptosis of a partial cells in response to *F. nucleatum* infection. *F. nucleatum*‐induced concomitant apoptosis and migration could be mutually exclusive process, and its net effect may be contingent on the cellular context and the specific state of the cells.

Genes such as N‐cadherin, Vimentin, SNAI1, and E‐cadherin are well‐recognized EMT markers, which were differentially expressed in *F. nucleatum*‐infected HIOECs and SCC‐9 cells (Fig. 4). SNAI1 is a prominent transcription factor regulating EMT and is capable of regulating the expression of N‐cadherin, Vimentin, and E‐cadherin 37. E‐cadherin plays a vital role in maintaining epithelial cell adhesion, and the abnormal expression and/or loss of its function is a major contributor to cancer progression 38. E‐cadherin loss is associated with increased cell motility, which explains the phenomenon that *F. nucleatum* promoted cell migration in our results.

FadA is a surface adhesin expressed by *F. nucleatum*, and Rubinstein *et al*. 6 found that FadA could bind to E‐cadherin on CRC cells and activate the EMT process. Further study illustrated that FadA could upregulate the expression of the Wnt/β‐catenin modulator Annexin A1 through E‐cadherin 39. To characterize the bacterial components involved in the regulation of EMT markers, heat‐inactivated *F. nucleatum* and purified FadA were applied to stimulate the cells and the results showed that both heat‐inactivated *F. nucleatum* and purified FadA could facilitate EMT via the upregulation of SNAI1 (Fig. S5), which identify that live whole *F. nucleatum* was not required for EMT induction, and FadA played a major role in this process. Nonetheless, other virulence factors may also participate in this process and *F. nucleatum* inducing EMT may involve multiple pathways.

Epithelial–mesenchymal transition program can be regulated by multiple signal pathways, such as the TGF‐ß pathway, Wnt signaling pathway, and the Notch pathway 11. In this study, high‐throughput sequencing was performed, and these results found that MIR4435‐2HG was significantly upregulated in the *F. nucleatum*‐infected cells compared with the control cells (~ 6‐fold). Higher expression of MIR4435‐2HG was observed in colorectal and gastric epithelial cancer tissues, and knockdown of MIR4435‐2HG inhibited the migration and invasion of gastric cancer cells 19, 40, 41. Studies have revealed that increased MIR4435‐2HG expression might be a potential biomarker for the diagnosis and prognosis of colorectal cancer, correlated with lymphatic metastasis, and predict poor survival in patients with gastric cancer or breast cancer 19, 41, 42. However, a specific role for MIR4435‐2HG in bacterial infection or EMT in oral epithelial cells has not been identified. Here, we identified that *F. nucleatum* infection upregulated the expression of MIR4435‐2HG, and knockdown of MIR4435‐2HG significantly inhibited SNAI1 expression, which indicated that MIR4435‐2HG played a major role in initiating EMT. To the best of our knowledge, this is the first time that MIR4435‐2HG has been reported to be involved in *F. nucleatum* infection‐induced EMT in either a non‐neoplastic or neoplastic oral epithelial cells.

Recently, lncRNAs have been proposed to act as miRNA sponges or competitive endogenous RNAs, forming extensive regulatory networks, thereby negatively regulating miRNA expression 43. For instance, lncRNA GAS5 suppresses proliferation, migration, invasion, and EMT in OSCC by regulating the miR‐21/PTEN axis 44. MiR‐296‐5p has been demonstrated to play important roles in various cellular processes, including cell proliferation, apoptosis, invasion, and EMT 45. Our results showed that miR‐296‐5p was prominently downregulated in *F. nucleatum*‐infected HIOECs and SCC‐9 cells (Fig. S9 and Fig. 6E), and knockdown of MIR4435‐2HG significantly increased the expression level of miR‐296‐5p. Moreover, the reduction in SNAI1 induced by knockdown of MIR4435‐2HG could be rescued by miR‐296‐5p inhibitor, and a luciferase reporter assay confirmed the direct binding of MIR4435‐2HG and miR‐296‐5p (Fig. 7B).

However, miR‐296‐5p did not bind the 3′UTR of SNAI1, as evidenced by the luciferase reporter assay (Fig. S10), which indicated that miR‐296‐5p might indirectly regulate SNAI1. In view of the above results, we speculated that a gene, which is a target gene of miR‐296‐5p and regulate SNAI1 simultaneously, may be involved in this process. Ultimately, we identified Akt2 as the candidate gene. Akt2 is a conserved serine/threonine kinase of the AKT family of proteins that are activated by the phosphatidylinositol‐3 kinase pathway. Increasing evidence has suggested that Akt2 regulates multiple processes, including metabolism, proliferation, cell survival, angiogenesis, and EMT as an oncogene 46. It has been previously demonstrated that Akt2 is a direct target of miR‐296‐5p and activates SNAI1 47, 48. In our study, Akt2 was upregulated in both *F. nucleatum*‐infected and miR‐296‐5p inhibitor‐transfected HIOECs and SCC‐9 cells and was downregulated in cells with MIR4435‐2HG knockdown, whereas Akt2 knockdown inhibited the expression of SNAI1 (Fig. 8).

Taken together, our study confirmed that *F. nucleatum* is able to trigger EMT in both normal and cancerous oral epithelial cells through the regulation of MIR4435‐2HG/miR‐296‐5p/Akt2/SNAI1 signaling pathway. It is noteworthy that EMT process has been well described in various carcinomas of epithelial origin and regarded to be associated with initiation of tumor. Therefore, our data can be considered as a preliminary evidence that EMT can be a link between *F. nucleatum* infection and initiation of oral epithelial carcinomas. Furthermore, it was inferred that *F. nucleatum* promoting EMT in both non‐neoplastic and cancer cells were independent of the viable whole bacterial cells and that FadA may be closely related to this process. Further studies are still warranted to decipher the specific bacterial effectors of *F. nucleatum* and the host–pathogen interactions involved in the EMT program.

## Materials and methods

### Hematoxylin–eosin staining and fluorescence in situ hybridization

Paraffin‐embedded OSCC and healthy tissue samples were cut into 4 μm sections and were subjected to HE staining according to a protocol previously described 49. The tissues were then applied to detect *F. nucleatum* by FISH as described previously 50. Briefly, the sections were deparaffinized by xylene and followed by sequential immersion in 100%, 95%, 70%, and 50% ethanol (5 min each). Then, the slides were incubated with permeabilization buffer containing lysozyme (10 mg·mL^−1^) at 37 °C for 20 min, and washed with PBS. The sections were treated with permeabilization buffer with proteinase K (7 μg·mL^−1^) at 37 °C for 20 min. Next, the prewarmed hybridization buffer without *F. nucleatum* probe was added and incubated at 48 °C for 20 min. Subsequently, the slides were incubated with hybridization buffer containing *F. nucleatum* oligonucleotide probe (0.1 μm) in a dark humid chamber at 48 °C for 1 h. Before fluorescence imaging, the sections were counterstained with DAPI for 2 min. The 5′ Alexa Flour 488‐labeled *F. nucleatum* probe was synthesized by Sangon (Shanghai, China), and the sequence was as follows: 5′‐CGCAATACAGAGTTGAGCCCTGC‐3′. The mounted slides were observed and photographed using the microscope (Nikon 80i, Tokyo, Japan). Procedures were approved by the ethics committee of School and Hospital of Stomatology, China Medical University. Written informed consent was obtained from all patients.

### Bacteria and cell culture

*Fusobacterium nucleatum* ATCC 25586 was inoculated on trypticase soy broth (TSB) agar plates supplemented with 5 μg·mL^−1^ hemin, 5% defibrinated sheep blood, 0.5% yeast extract, and 1 μg·mL^−1^ vitamin K1 in an anaerobic chamber for 2–3 days. *S. gordonii* Challis CH1 was cultured aerobically at 37 °C in TSB with 0.5% yeast extract. For *F. nucleatum* inactivation, the bacteria were heat‐inactivated at 60 °C for 60 min in a water bath as described previously 51. Killing was confirmed by absence of growth on blood agar plates. HIOECs, generously provided by Key Laboratory of Shanghai Oral Medicine, Shanghai Jiao Tong University, were cultured in defined keratinocyte serum‐free medium (Gibco, Thermo Fisher Scientific Inc., Waltham, MA, USA) with growth factor supplement. Two human squamous cell carcinoma cell lines SCC‐9 and HSC‐4 were used in this study. SCC‐9 (ATCC CRL‐1629) from American Type Culture Collection (Manassas, VA, USA) was grown in DMEM/Ham's F‐12 medium supplemented with 10% fetal bovine serum (Biological Industries, Kibbutz, Israel) and 400 ng·mL^−1^ hydrocortisone (Sigma‐Aldrich, St Louis, MO, USA). HSC‐4 cells were purchased from the Cell Bank of Japanese Collection of Research Bioresource (JCRB, Shinjuku, Japan) and cultured in DMEM/high glucose (Hyclone, Logan, UT, USA) containing 10% fetal bovine serum. All cell lines were grown at 37 °C with 5% CO2 in a humidified atmosphere. For all the experiments in this study, the bacteria were added to the cells at a MOI of 100 : 1 based on the preliminary experiments.

### Cell proliferation assay

Human immortalized oral epithelial cells, SCC‐9, and HSC‐4 cells were seeded at 5 × 10^3^ cells per well in 96‐well plates and infected with *F. nucleatum* or *S. gordoni*i at a MOI of 100 : 1. Cells without *F. nucleatum* or *S. gordonii* infection were used as the negative control. At each time point, the medium was removed, and 100 μL basic medium containing 10% CCK‐8 reagent (Dojindo, Kumamoto, Japan) was added and incubated for 1.5 h. The optical density was determined at 450 nm using a microplate reader (Tecan, Groedig, Austria).

### Cell cycle and cell apoptosis

Human immortalized oral epithelial cells and SCC‐9 were seeded in 6‐well plates at 2.5 × 10^5^ cells per well and infected with *F. nucleatum* at a MOI of 100 : 1 for 24 h. For cell cycle analysis, HIOECs and SCC‐9 were synchronized by serum starvation for 24 h before *F. nucleatum* infection. The cells were digested with EDTA‐trypsin and fixed in 70% ethanol for 2 h or longer at 4 °C. Then, the cells were incubated with PI and RNase A (Beyotime Biotech. Co., Shanghai, China) for 15 min at room temperature prior to flow cytometer detection. For apoptosis detection, HIOECs and SCC‐9 cells infected with *F. nucleatum* or heat‐inactivated *F. nucleatum* and control cells were collected by trypsin without EDTA and then assayed using the Annexin V‐PI staining kit (eBioscience, San Diego, CA, USA) in accordance with the manufacturer's instructions. The cells were analyzed by flow cytometer (FACS, BD, Franklin Lake, NJ, USA).

### Cell migration assay

Human immortalized oral epithelial cells, SCC‐9, and HSC‐4 cells were grown to 80–90% confluence and pretreated with mitomycin‐C (Sigma‐Aldrich) for 1 h before *F. nucleatum* or *S. gordonii* infection (MOI = 100 : 1). A wound was created using a yellow pipette tip, the cells were gently washed twice with PBS, and cell motility was assayed by measuring the average scratch width change. Images were captured at regular time intervals, the healing areas of specified scratches were assessed using image‐pro plus software (version 6.0; Media Cybernetics, Inc., Rockville, MD, USA), and the healing rate was calculated as (%) = [(initial average scratch width at 0 h − average scratch width at 24 h)/initial average scratch width × 100%] 52.

### Gelatin zymography assay

The activities of MMP2 and MMP9 in supernatant of HIOECs, SCC‐9, and HSC‐4 cells infected with *F. nucleatum* at a MOI of 100 : 1 as well as noninfected controls were determined using gelatin zymography as described 53. In brief, the protein concentration was measured using a BCA protein assay kit (Beyotime Biotech. Co.). Then, the samples containing equal amounts of proteins were mixed with 2× SDS/PAGE nonreducing buffer and separated on 8% SDS/PAGE gels containing 0.1% gelatin. The gels were then incubated with 1× Buffer A for 4 h at room temperature and incubated in 1× Buffer B of the MMP Zymography Assay Kit (Applygen Technologies Inc., Beijing, China) for 48 h at 37 °C. After staining with 0.5% Coomassie brilliant blue R‐250, the gels were incubated in a destaining solution for 2‐4 h. The gelatinase activities were visualized as clear bands against a blue background and quantified using imagej.

### Western blot analysis

Human immortalized oral epithelial cells, SCC‐9, and HSC‐4 cells were lysed using RIPA buffer containing protease inhibitors on ice, and total protein was quantified using a BCA Protein Assay Kit (Beyotime Biotech. Co.). Equal amounts of protein were loaded onto 10% SDS/PAGE gels and transferred to nitrocellulose membranes. Then, the membranes were blocked with 5% skimmed milk in Tris‐buffered saline containing 0.1% Tween‐20 (TBST) and incubated with primary antibodies overnight at 4 °C, followed by secondary antibody incubation. The antibodies used in this study include anti‐E‐cadherin (1 : 5000; Abcam, Cambridge, MA, USA), anti‐Vimentin (1 : 500; Abcam), anti‐SNAIL1 (1 : 1000; Cell Signaling Technology, Danvers, MA, USA), anti‐N‐cadherin (1 : 1000; Cell Signaling Technology), anti‐Akt2 (1 : 1000; Cell Signaling Technology), and anti‐α‐tubulin (1 : 2000; Beyotime Biotech. Co.). Images were acquired by Odyssey CLX (LI‐COR, Lincoln, NE, USA), and band analyses were performed using imagej software (NIH).

### Immunofluorescent staining

Both HIOECs and SCC‐9 were plated onto slides in 12‐well plates and infected with *F. nucleatum* at a MOI of 100 : 1. Both cells were fixed with 4% paraformaldehyde for 30 min at 4 °C and then permeabilized with 0.1% Triton X‐100 in PBS for 5 min. After being washed three times in PBS and blocked with 1% BSA for 1 h at room temperature, cells were incubated with the primary antibodies (anti‐E‐cadherin, anti‐N‐cadherin, anti‐Vimentin, and anti‐SNAIL1, 1 : 200 dilution in PBS) overnight at 4 °C. Then, the cells were incubated with Alexa Fluor 488‐ or 594‐conjugated secondary antibodies (1 : 200 dilution; Proteintech Group, Chicago, IL, USA) for 1 h at room temperature. HIOECs and SCC‐9 cells were counterstained with DAPI (Beyotime Biotech. Co.) before fluorescence microscopy and image capture (Nikon 80i). Positive staining cells were analyzed for the intensity of fluorescence using imagej software (NIH).

### Purification of recombinant FadA protein

*Escherichia coli* carrying the entire FadA gene in an expression vector was used to produce and purify FadA as previously described 54.

### High‐throughput sequencing and data analysis

Total RNA from HIOECs and *F. nucleatum*‐infected HIOECs was extracted using TRIzol (Invitrogen, Carlsbad, CA, USA) and applied for lncRNA high‐throughput sequencing on an Illumina HiSeq3000 sequencer (Illumina, San Diego, CA, USA). The sequencing analysis, including labeling, hybridization, scanning, normalization, and data analysis, was performed by Genergy Biotechnology (Shanghai, China). The criteria including |Log2FC| > 2, *P* < 0.05, and FDR < 0.05 were applied to select the significantly differentially expressed lncRNAs and mRNAs. Some EMT‐related genes were clustered with multi experiment viewer (mev, version 4.6.0, JCVI, La Jolla, CA, USA). KEGG pathway analysis (http://www.genome.jp/kegg/) was further applied to define the functions of the selected genes.

### Gene expression analysis by qRT‐PCR

Total RNA was extracted using TRIzol reagent, and cDNA was synthesized using the RR047A kit or miRNA First Strand cDNA Synthesis (tailing reaction) kit according to the manufacturer's instructions. qRT‐PCR was carried out in an Applied Biosystems 7500 Real‐Time PCR System (Waltham, MA, USA) with the RR820A kit. The reagents were purchased from Takara (Kyoto, Japan) and Sangon Biotech (Shanghai, China). The primers are listed in Table S1. β‐actin and U6 were selected as the endogenous controls, and the $2^{-\Delta \Delta C_{T}}$ method was used to analyze the relative gene expression data.

### Binding sites prediction and bioinformatics analysis

The subcellular localization of MIR4435‐2HG was searched in lncATLAS (lncatlas.crg.eu) and the published literature, and the results showed that MIR4435‐2HG was mainly expressed in the cytoplasm. The trans‐target genes regulated by MIR4435‐2HG were predicted by coexpression analysis. The Pearson correlation coefficient was used, and *R* ^2^ ≥ 0.95 was set as the screening criterion. The coexpression of MIR4435‐2HG and SNAI1 in HNSCC was searched on TCGA‐Pan‐Cancer (ChIPBase v2.0). DIANA Tools (LncBase v.2) and miRDB (http://www.mirdb.org/) were utilized to predict the interacting microRNAs.

### Cells transfection

siRNAs with chemical modifications that targeted MIR4435‐2HG, scrambled siRNA control (si‐NC), miR‐296‐5p inhibitor (miR‐inhibitor), and its negative control (miR‐NC) were designed and synthesized by GenePharma Co. Ltd. (Shanghai, China), and the sequences are listed in Table S2. To investigate the effect of MIR4435‐2HG on SNAI1 and miR‐296‐5p expression, HIOECs and SCC‐9 cells were transfected with siRNAs targeting MIR4435‐2HG or si‐NC via Golden Tran^®^‐R (Golden Transfer Science and Technology Co. Ltd., Changchun, China) according to the manufacturer's instructions. The transfected HIOECs and SCC‐9 cells were subsequently infected with *F. nucleatum*. To evaluate the relationship between MIR4435‐2HG and miR‐296‐5p and the regulation of miR‐296‐5p on SNAI1, siRNAs targeting MIR4435‐2HG and miR‐inhibitor were transfected into HIOECs and SCC‐9 cells individually or together. The regulation of Akt2 on SNAI1 was confirmed via Akt2 knockdown by siRNA targeting Akt2. The specific silencing of MIR4435‐2HG, miR‐296‐5p, and Akt2 expression was assessed by qRT‐PCR. Cells were harvested at 48 or 72 h after transfection for subsequent qRT‐PCR or western blot analysis, respectively.

### Dual‐luciferase reporter assay

The binding sites of miR‐296‐5p in MIR4435‐2HG and SNAI1 were predicted by RNAhybrid 2.2 (http://bibiserv.techfak.uni-bielefeld.de/rnahybrid/) and miRDB, respectively. MIR4435‐2HG and SNAI1 3′‐UTR fragments containing the predicted binding site of miR‐296‐5p were amplified and inserted into pmirGlo vectors to construct wild‐type plasmids MIR4435‐2HG‐wt and SNAI1‐wt, as well as mutant‐type plasmids MIR4435‐2HG‐mut and SNAI1‐mut. The plasmids were synthesized by GenePharma Co. Ltd. HEK 293T cells were cotransfected with the luciferase plasmids together with miR‐296‐5p mimics or mimics‐NC using Lipofectamine 2000 (Invitrogen). At 48 h posttransfection, luciferase activity was measured using the Dual‐Luciferase Reporter Assay System (Promega, Madison, WI, USA) in accordance with the manufacturer's instructions. Renilla luciferase activity was used for normalization. All assays were performed in triplicate with three independent experiments (*n* = 3).

### Statistical analysis

The data were presented as the mean ± standard deviation (SD) of at least three independent experiments (*n* = 3), and statistical analyses were performed using spss 22.0 (SPSS, Inc., Chicago, IL, USA). Student's *t‐*test was performed for a single comparison between two groups, and one‐way analysis of variance (ANOVA), followed by Student–Newman–Keuls multiple comparisons, was applied for a comparison between multiple groups. A *P* < 0.05 was considered statistically significant. graphpad prism (San Diego, CA, USA) software was used to generate graphs.

## Conflict of interest

The authors declare no conflict of interest.

## Author contributions

YP, CL, and SZ designed the study. SZ performed the experiments with the help from YP, CL, JL, FG, QL, XS, and ZL. SZ wrote the final manuscript. YP and CL revised the manuscript. All authors read and approved the final manuscript.

## Supporting information


**Fig. S1.** Effect of *F. nucleatum* infection on cell cycle progression and apoptosis.
**Fig. S2.** Culture supernatants were analyzed for MMP‐9 and MMP‐2 activities by gelatin zymography.
**Fig. S3.** SNAI1 mRNA levels in HIOECs after 24 h infection with *F. nucleatum* at MOI indicated.
**Fig. S4.** *F. nucleatum* but not *S. gordonii* significantly induced SNAIL1 protein expression.
**Fig. S5.** FadA and heat‐inactivated *F. nucleatum* induce the expression of EMT markers.
**Fig. S6.** ZEB2, TWIST1, and SLUG mRNA levels were measured following *F. nucleatum* infection for 24 h.
**Fig. S7.** Differentially expressed genes identified by high‐throughput sequencing were validated in *F. nucleatum*‐infected HIOECs and SCC‐9 cells by qRT‐PCR.
**Fig. S8.** The expression of MIR4435‐2HG and SNAI1 were positively correlated in head and neck squamous cell carcinoma based on the TCGA‐Pan‐Cancer (ChIPBase v2.0) database.
**Fig. S9.** The expression of miRNAs predicted by an online database were quantified by qRT‐PCR in *F. nucleatum*‐infected HIOECs.
**Fig. S10.** The dual‐luciferase reporter assay of HEK 293T cells cotransfected with SNAI1‐wt or SNAI1‐mut and miR‐296‐5p mimics or mimics‐NC.
**Table S1.** Primer sequences for qRT‐PCR used in this study.
**Table S2.** The sequences of the oligos used in this study.
