# Supplementary material for: Fusobacterium nucleatum promotes epithelial‐mesenchymal transiton through regulation of the lncRNA MIR4435‐2HG/miR‐296‐5p/Akt2/SNAI1 signaling pathway
Source: FEBS J. 2020 Feb 12;287(18):4032–47. doi: 10.1111/febs.15233 (PMC7540502; doi:10.1111/febs.15233)
Supplement: Supplementary file 1 — Fig. S1. Effect of F. nucleatum infection on cell cycle progression and apoptosis. Fig. S2. Culture supernatants were analyzed for MMP‐9 and MMP‐2 activities by gelatin zymography. Fig. S3. SNAI1 mRNA levels in HIOECs after 24 h infection with F. nucleatum at MOI indicated. Fig. S4. F. nucleatum but not S. gordonii significantly induced SNAIL1 protein expression. Fig. S5. FadA and heat‐inactivated F. nucleatum induce the expression of EMT markers. Fig. S6. ZEB2, TWIST1, and SLUG mRNA levels were measured following F. nucleatum infection for 24 h. Fig. S7. Differentially expressed genes identified by high‐throughput sequencing were validated in F. nucleatum‐infected HIOECs and SCC‐9 cells by qRT‐PCR. Fig. S8. The expression of MIR4435‐2HG and SNAI1 were positively correlated in head and neck squamous cell carcinoma based on the TCGA‐Pan‐Cancer (ChIPBase v2.0) database. Fig. S9. The expression of miRNAs predicted by an online database were quantified by qRT‐PCR in F. nucleatum‐infected HIOECs. Fig. S10. The dual‐luciferase reporter assay of HEK 293T cells cotransfected with SNAI1‐wt or SNAI1‐mut and miR‐296‐5p mimics or mimics‐NC. Table S1. Primer sequences for qRT‐PCR used in this study. Table S2. The sequences of the oligos used in this study. [file FEBS-287-4032-s001.zip › febs15233-sup-0001-Supinfo.pdf]

FEBS PRESS
science publishing by scientists
WILEY
The FEBS Journal
www.febsjournal.org

Fusobacterium nucleatum promotes epithelial-mesenchymal transiton through regulation of the lncRNA MIR4435-2HG/miR-296-5p/Akt2/SNAI1 signaling pathway

Shuwei Zhang, Chen Li, Junchao Liu, Fengxue Geng, Xiaoting Shi, Qian Li, Ze Lu and Yaping Pan

DOI: 10.1111/febs.15233

Fusobacterium nucleatum Promotes Epithelial-Mesenchymal Transition through Regulation of the lncRNA MIR4435-2HG/miR-296-5p/Akt2/SNAI1 Signaling Pathway

Shuwei Zhang[1], Chen Li[1,2], Junchao Liu[1], Fengxue Geng[1], Xiaoting Shi[1], Qian Li[2], Ze Lu[1], Yaping Pan[1,2]*

[1]Department of Periodontics, School and Hospital of Stomatology, China Medical University

[2]School and Hospital of Stomatology, China Medical University, Liaoning Provincial Key Laboratory of Oral Diseases

*Correspondence:

Yaping Pan

Department of Periodontics, School and Hospital of Stomatology, China Medical University, Shenyang, 110002, China.

Telephone: +86-24-31927706

Email: yppan@cmu.edu.cn

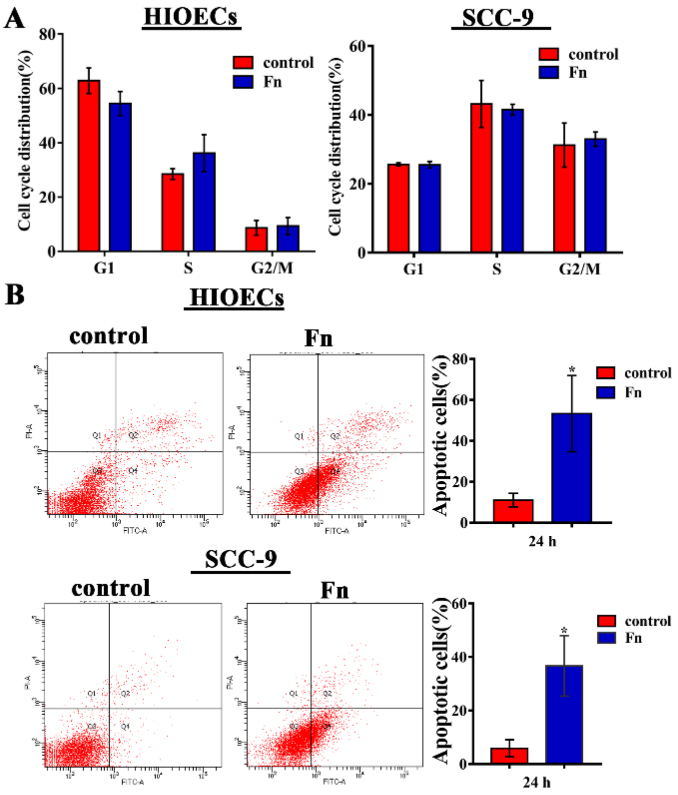

Supplementary Figure S1. Effect of *F. nucleatum* infection on cell cycle progression and apoptosis. HIOECs and SCC-9 cells were infected with *F. nucleatum* at a MOI of 100:1 for 24 h, and (**A**) The cell cycle distribution and (**B**) apoptosis were determined by flow cytometry-based assay and analyzed with ModiFit software. The data are presented as the mean ± standard deviation (SD) obtained from three independent experiments (n=3). * *P*<0.05, ** *P*<0.01 vs. the control cells (Student's *t* test).

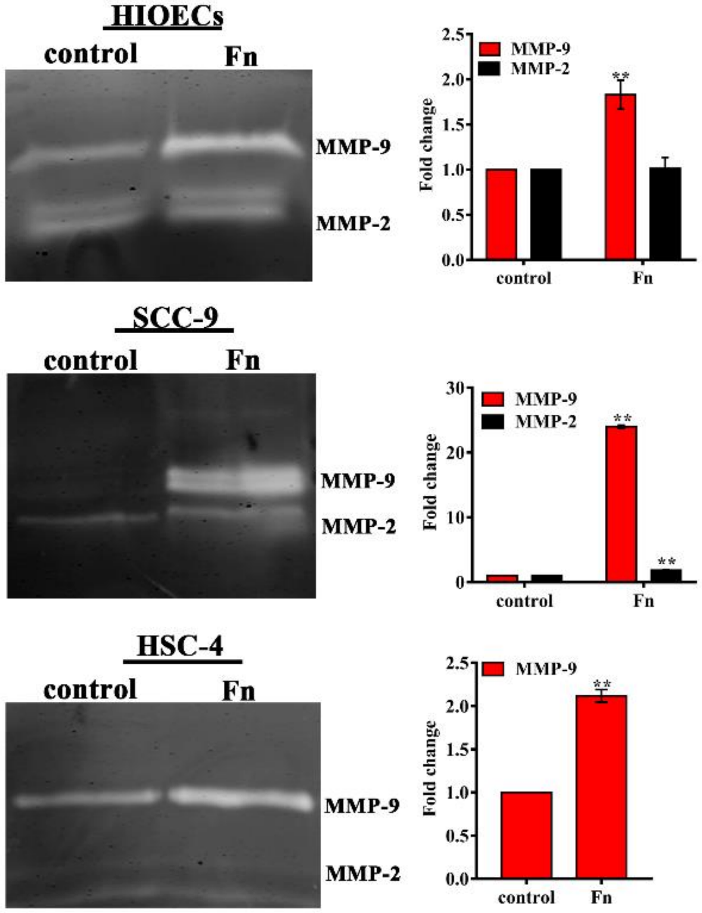

Supplementary Figure S2. Culture supernatants were analyzed for MMP-9 and MMP-2 activities by gelatin zymography. MOI=100:1. MMP-9 secretion was clearly elevated in the culture medium following *F. nucleatum* infection, while there was no stimulation of MMP-2. The data are presented as the mean ± standard deviation (SD) obtained from three independent experiments (n=3). * P<0.05, ** P<0.01 vs. the control cells (Student's t test).

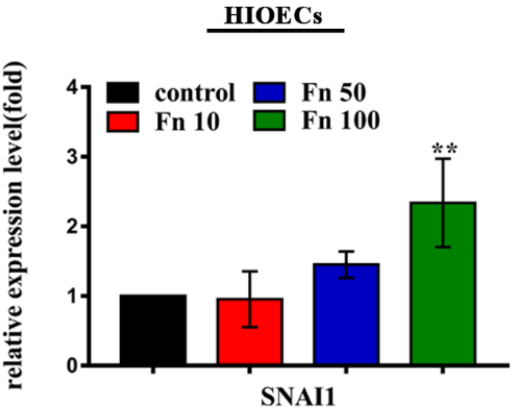

Supplementary Figure S3. SNAI1 mRNA levels in HIOECs after 24 h infection with *F. nucleatum* at MOI indicated. qRT-PCR data were normalized to ß-actin. The data are presented as the mean ± standard deviation (SD) obtained from three independent experiments (n=3). ** *P*<0.01 vs. the control cells (Student's *t* test).

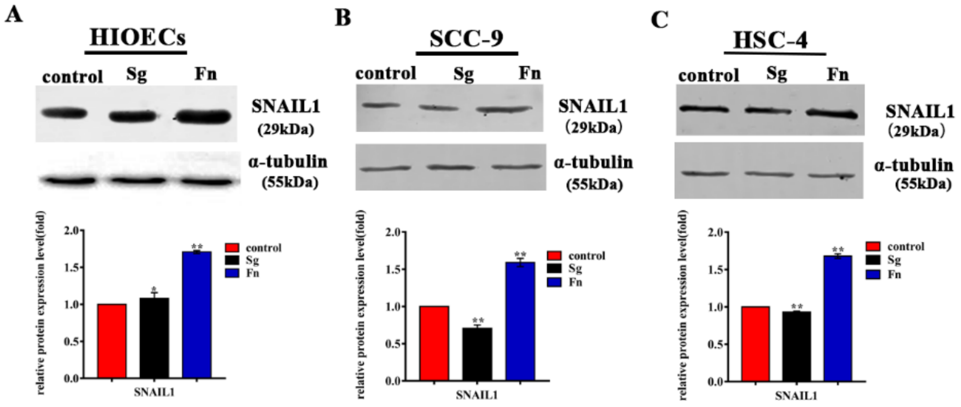

Supplementary Figure S4. *F. nucleatum* but not *S. gordonii* significantly induced SNAIL1 protein expression. (**A, B, C**) The three cell lines were infected with *F. nucleatum* or *S. gordonii* at a MOI of 100:1 and SNAIL1 protein were determined by western blot. The data are presented as the mean ± standard deviation (SD) obtained from three independent experiments (n=3). * *P*<0.05, ** *P*<0.01 vs. the control cells (Student's *t* test)).

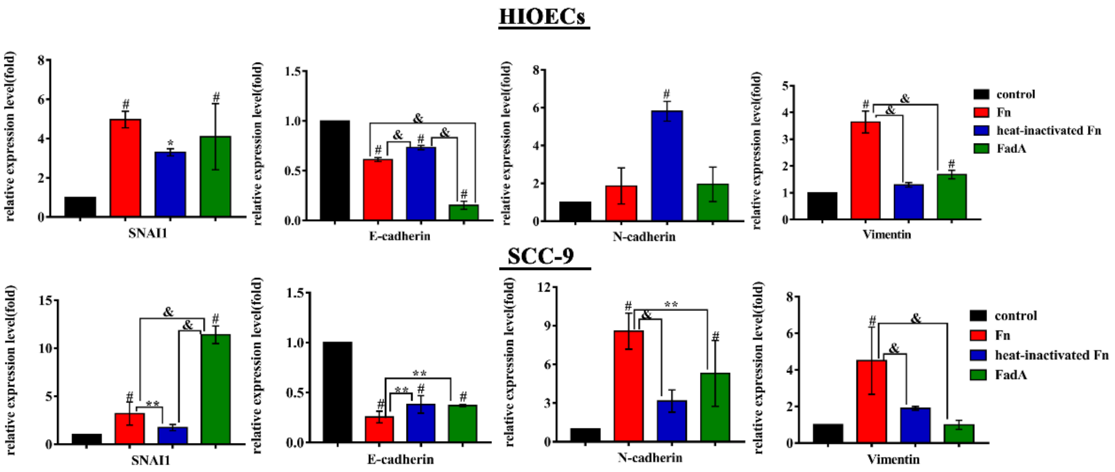

Supplementary Figure S5. FadA and heat-inactivated *F. nucleatum* induce the expression of EMT markers. MOI=100:1. The data are presented as the mean ± standard deviation (SD) obtained from three independent experiments (n=3). * $P<0.05$, # $P<0.01$ vs. control cells; & $P<0.01$, ** $P<0.05$ for comparisons among Fn, heat-inactivated Fn and FadA groups (one-way ANOVA).

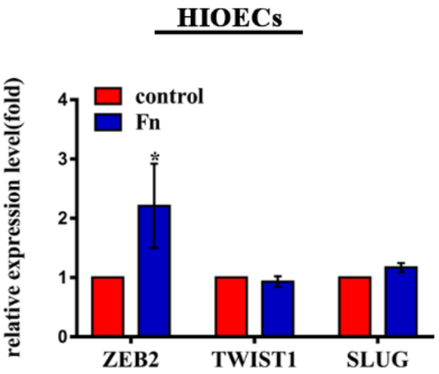

Supplementary Figure S6. ZEB2, TWIST1, and SLUG mRNA levels were measured following *F. nucleatum* infection for 24 h. MOI=100:1. The data are presented as the mean ± standard deviation (SD) obtained from three independent experiments (n=3). * $P<0.05$ vs. the control cells (Student's *t* test).

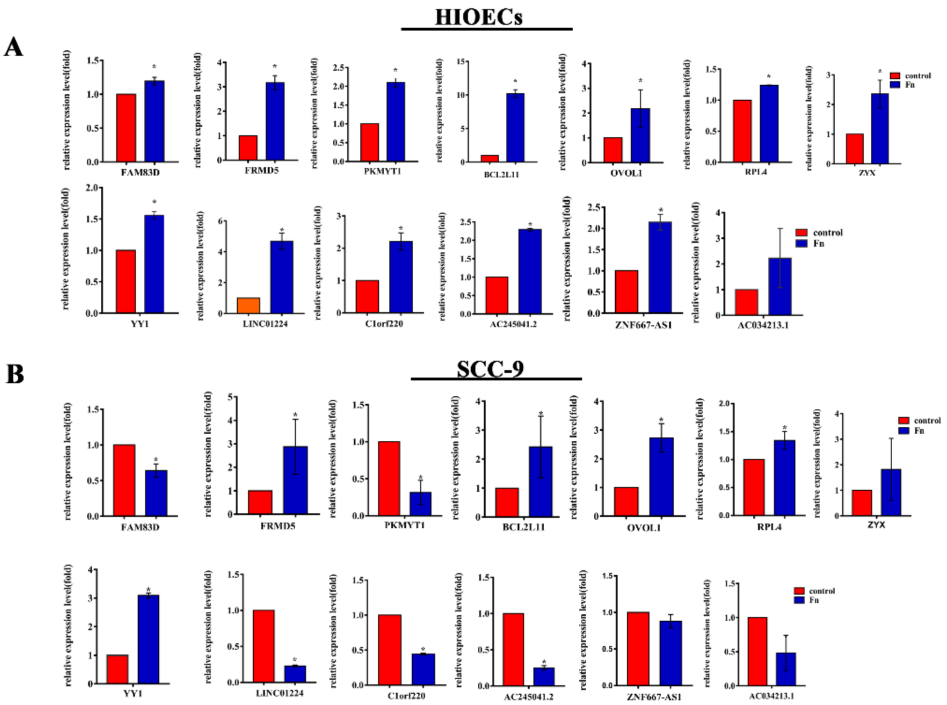

Supplementary Figure S7. Differentially expressed genes identified by high-throughput sequencing were validated in *F. nucleatum*-infected HIOECs and SCC-9 cells by qRT-PCR. MOI=100:1. The data are presented as the mean ± standard deviation (SD) obtained from three independent experiments (n=3). * P<0.05 vs. control cells (Student's t test).

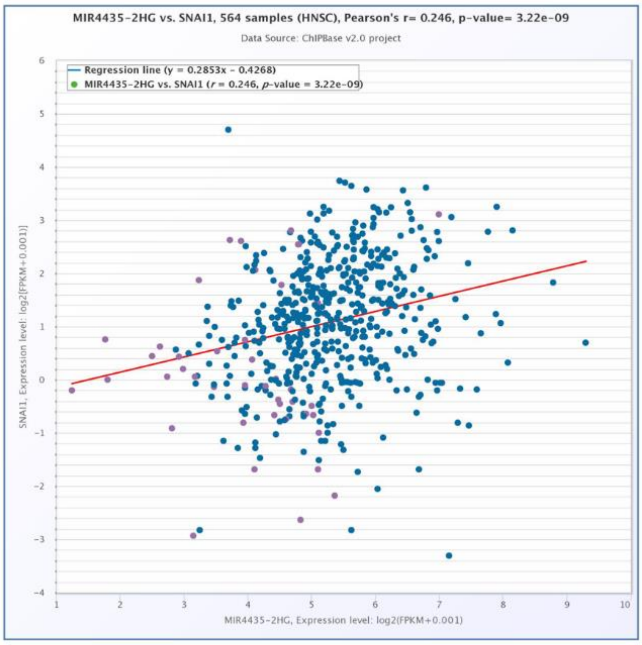

Supplementary Figure S8. The expression of MIR4435-2HG and SNAI1 were positively correlated in head and neck squamous cell carcinoma based on the TCGA-Pan-Cancer (ChIPBase v2.0) database.

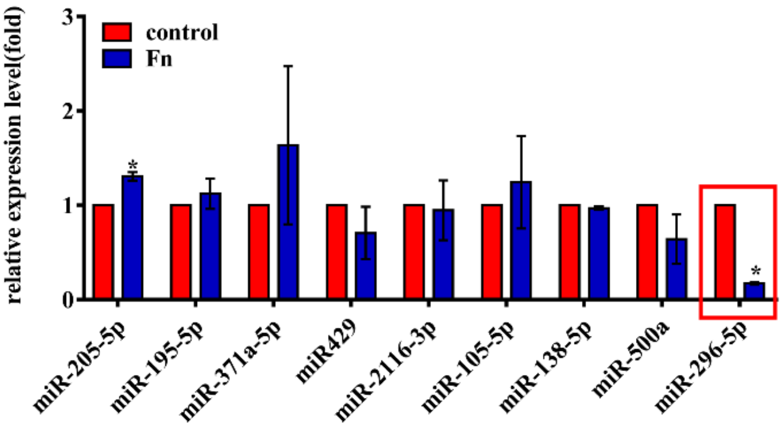

Supplementary Figure S9. The expression of miRNAs predicted by an online database were quantified by qRT-PCR in *F. nucleatum*-infected HIOECs. MOI=100:1. Only miR-296-5p was decreased significantly compared with that in the control cells. The data are presented as the mean ± standard deviation (SD) obtained from three independent experiments (n=3). * P<0.05 vs. control cells (Student's t test).

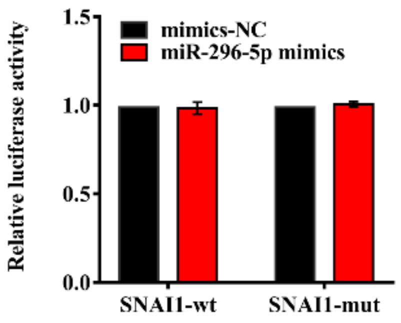

Supplementary Figure S10. The dual-luciferase reporter assay of HEK 293T cells cotransfected with SNAI1-wt or SNAI1-mut and miR-296-5p mimics or mimics-NC. The data are presented as the mean ± standard deviation obtained from three independent experiments (n=3) (No significant by Student's *t* test).

Supplementary Table S1. Primer sequences for qRT-PCR used in this study.

Gene	Forward sequence	Reverse sequence
E-cadherin	CCTGGGACTCCACCTACAGAA	AGGAGTTGGGAAATGTGAGC
Vimentin	AGGCGAGGAGAGCAGGATTT	AGTGGGTATCAACCAGAGGGA
SNAI1	CCAGTGCCTCGACCACTATG	GCAGCTCGCTGTAGTTAGGCTTC
N-cadherin	AACAGCAACGACGGGTTAGT	CAGACACGGTTGCAGTTGAC
YY1	CCTCTCAGATCCCAAACAACTG	GCCTTTATGAGGGCAAGCTATT
FRMD5	CTCCAACTCCTGAAGCGTGTAAGC	GCTGGACACTGTGCGGACTTG
FAM83D	GGGAAGGTTCACGAAAAGTTCA	GACTGGGCATACAGGATTCGG
PKMYT1	CATGGCTCCTACGGAGAGGT	ACATGGAACGCTTTACCGCAT
ZYX	GTACTGCGAGGGCTGTTACAC	GGGCGTACTGCTTGTGGTA
RPL4	TGTTTGCACCAACCAAAACCT	GCAGAACAGATGGCGTATCGT
OVOL1	GAACATGAGCCTTCGAGACTCTAGC	GCAGACACGGCAGGTGAACAG
MMP2	GACATACATCTTTGCTGGAGAC	TTCAGGTAATAGGCACCCTT
MMP3	GGTGTGGAGTTCCTGATGTTGGTC	AGCCTGGAGAATGTGAGTGGAGTC
MMP9	CTTCACTTTCCTGGGTAAGG	CACTTCTTGTCGCTGTCAAA
MMP13	CCTGGCTGCCTTCCTCTTCTTG	GCCTCTCAGTCATGGAGCTTGC
BCL2L11	TAAGTTCTGAGTGTGACCGAGA	GCTCTGTCTGTAGGGAGGTAGG
MIR4435-2HG	CATCCACATTCCAACCTCCGTCTG	GCAGGACAGAGGCAGCTTAGAATC

ZNF667-AS1	AGGTGACCGTTGCGTAATTGTGAG	CTCCACTGCCTGGAATGTGTCTG
C1orf220	CAGGACACGGACTCTTGAAGGTTG	CAGGAGGCAGCACCACATTCAG
LINC01224	GCTATTCAACGTCCTCCACCTCTG	GTCCTGGCATCTTAGCTGTCTGTG
AC034213.1	GGTGTGGCTCTGGAGTTACGTG	TGCCTTCACGCGGCTGTAATC
AC245041.2	CTGCAGCCGTTTTCCAGACA	ATCGTGACAGCCCCTGCTTA
miR-296-5p	tAGGGCCCCCCCTCAATC	GTGCAGGGTCCGAGGT
Akt2	TCCGAGGTCGACACAAGGTA	CTGGTCCAGCTCCAGTAAGC
U6	GCGCGTCGTGAAGCGTTC	GTGCAGGGTCCGAGGT
β-actin	TGGCACCCAGCACAATGAA	CTAAGTCATAGTCCGCCTAGAAGCA

Supplementary Table S2. The sequences of the oligos used in this study.

Name	Sequences
siRNA-1 for MIR4435-2HG	Forward: 5’-GGUCACUACU GCUUUAUAATT-3'
	Reverse: 5’-UUAUAAAGCAGUAGUGACCTT-3’
siRNA-2 for MIR4435-2HG	Forward:5’GCCCAGAUUUAAGGGCUAUTT-3’
	Reverse:5’-AUAGCCCUUAAAUCUGGGCTT-3’
siRNA-3 for MIR4435-2HG	Forward: 5’-CCAGUCUCUGUGUGUCUUATT-3’
	Reverse: 5’-UAAGACACACAGAGACUGGTT-3’
siRNA for Akt2	Forward:5’-AAGGAUGAAGUCGCUCACACA-3’
	Reverse:5’-UGUGUGAGCGACUUCAUCCUU-3’
siRNA-NC	Forward: 5’-UUCUCCGAACGUGUCACGUTT-3’
	Reverse: 5’-ACGUGACACGUUCGGAGAATT-3’
miR-296-5p inhibitor	5’-ACAGGAUUGAGGGGGGGCCCU-3’
miR-NC	5’-CAGUACUUUUGUGUAGUACAA-3’
miR-296-5p mimics for luciferase reporter assay	Forward:5’-AGGGCCCCCCCUCAAUCCUGU-3’
	Reverse:5’-AGGAUUGAGGGGGGGCCCUUU-3’
mimics-NC for luciferase reporter assay	Forward: 5’-UUCUCCGAACGUGUCACGUTT-3’
	Reverse:5’-ACGUGACACGUUCGGAGAATT-3’
